# Expectations of Intensive Care Physicians Regarding an AI-Based Decision Support System for Weaning From Continuous Renal Replacement Therapy: Predevelopment Survey Study

**DOI:** 10.2196/63709

**Published:** 2025-04-23

**Authors:** Benjamin Popoff, Sandie Cabon, Marc Cuggia, Guillaume Bouzillé, Thomas Clavier

**Affiliations:** 1 Department of Anesthesiology, Critical Care and Perioperative Medicine, CHU Rouen, 37 Bd Gambetta, Rouen, 76000, France, 33 232888292; 2 Univ Rennes, CHU Rennes, INSERM, LTSI-UMR 1099, Rennes, France; 3 Normandie Univ, UNIROUEN, INSERM U1096, Rouen, France

**Keywords:** clinical decision support system, artificial intelligence, decision support, decision making, clinical decision making, survey study, intensive care physicians, renal replacement therapy, therapeutic, ICU, user-centered design, cross-sectional survey, survey, French, physician, questionnaire, AI tools, user-centered

## Abstract

**Background:**

Critically ill patients in intensive care units (ICUs) require continuous monitoring, generating vast amounts of data. Clinical decision support systems (CDSS) leveraging artificial intelligence (AI) technologies have shown promise in improving diagnostic, prognostic, and therapeutic decision-making. However, these models are rarely implemented in clinical practice.

**Objective:**

The aim of this study was to survey ICU physicians to understand their expectations, opinions, and level of knowledge regarding a proposed AI-based CDSS for continuous renal replacement therapy (CRRT) weaning, a clinical decision-making process that is still complex and lacking in guidelines. This will be used to guide the development of an AI-based CDSS on which our team is working to ensure user-centered design and successful integration into clinical practice.

**Methods:**

A prospective cross-sectional survey of French-speaking physicians with clinical activity in intensive care was conducted between December 2023 and April 2024. The questionnaire consisted of 20 questions structured around 4 axes: overview of the problem and current practices concerning weaning from CRRT, opinion on AI-based CDSS, implementation in daily clinical practice, real-life operation and willingness to adopt the CDSS in everyday practice. Statistical analyses included Wilcoxon rank sum tests for quantitative variables and *χ*^2^ or Fisher exact tests for qualitative variables, with multivariate analyses performed using ordinal logistic regression.

**Results:**

A total of 171 complete responses were received. Physicians expressed an interest in a CDSS for CRRT weaning, with 70.2% (120/171) viewing AI-based CDSS favorably. Opinions were split regarding the difficulty of the weaning decision itself, with 46.2% (79/171) disagreeing that it is challenging, while 31.6% (54/171) agreed. However, 66.1% (113/171) of respondents supported the value of an AI-based CDSS to assist them in this decision, with younger physicians showing stronger support (81.8%, 27/33 vs 62.3%; 86/138; *P*=.01). Most respondents (163/171, 95.3%) emphasized the importance of understanding the criteria used by the model to make its predictions.

**Conclusions:**

Our findings highlight an optimistic attitude among ICU physicians toward AI-based CDSS for CRRT weaning, emphasizing the need for transparency, integration into existing workflows, and alignment with clinicians’ decision-making processes. Actionable recommendations include incorporating key variables such as urine output and biological parameters, defining probability thresholds for recommendations and ensuring model transparency to facilitate the successful adoption and integration into clinical practice. The methodology of this survey may help the development of further predevelopment studies accompanying AI-based CDSS projects.

## Introduction

Critically ill patients admitted to intensive care units (ICUs) require continuous monitoring and surveillance of clinical, biological, and imaging parameters. This generates a large amount of data, making effective data exploitation a key challenge [1]. Recent advances in artificial intelligence (AI), particularly machine learning (ML), have facilitated the development of diagnostic, prognostic, and therapeutic decision-making aids known as clinical decision support systems (CDSS). These AI-based CDSS have demonstrated potential across several domains in ICU, including sepsis prediction, early detection of patient deterioration, and management of mechanical ventilation [2-4]. For instance, AI models for sepsis prediction can alert clinicians to septic events hours before clinical signs emerge, enabling timely interventions [5]. Similarly, early warning systems for patient deterioration, have shown promise in improving outcomes through real-time data analysis [2].

Despite these advances, most AI-based CDSS models fail to progress beyond the prototype stage and are rarely deployed into clinical practice, even though their performance is sometimes promising [6]. A recent meta-analysis analyzed publications concerning the use of AI in the ICU: out of the 494 articles studied, the majority were retrospective studies 476 articles (96.4%). In total, 10 articles (10/494, 2%) were clinical studies, of which only 5 (5/494, 1%) were randomized trials with a control arm. In addition, the majority of retrospective studies presented a high risk of bias [3]. In the domain of renal care we also observe this disconnect, where AI algorithms have been developed to predict acute kidney injury (AKI), offering valuable insights into early identification and prognosis [7,8]. AKI, a common condition affecting up to 60% of ICU patients [9], often necessitates renal replacement therapy (RRT) in severe cases. Continuous renal replacement therapy (CRRT) is a specific modality of RRT frequently used in critically ill patients, providing continuous 24-hour support that is well-suited for hemodynamically unstable patients [10].

While significant research has focused on the optimal timing for initiating CRRT [11,12], much less attention has been given to the question of when and how to discontinue this therapy [13]. This represents a critical gap in clinical practice, as premature weaning may lead to the recurrence of renal dysfunction, associated with complications such as fluid overload and electrolyte imbalances necessitating the reintroduction of renal support [14,15]. On the other hand, unnecessary prolongation of therapy can expose patients to greater risks, including catheter-related complications, higher infection rates, unnecessary resource utilization, extended ICU stays and delayed renal recovery [16]. Thus, identifying reliable criteria and developing decision support tools for successful weaning from CRRT is an ongoing and essential area of research.

AI-based CDSS present a compelling solution to this problem. By leveraging ICU data, these tools could provide individualized weaning recommendations and support clinicians in navigating the complex factors that influence this decision. However, to ensure successful adoption, it is crucial to align the development of these tools with end-user expectations and clinical realities. Obstacles to the development of mature AI models in routine ICU care arise at different stages of the CDSS development cycle. In particular in the predevelopment phase, with issues of privacy, representativeness, and complexity of heterogeneous ICU data, and in the postdevelopment phase (real-life deployment), with concerns about interoperability and end-user usability [6,17]. Recent frameworks have been proposed to ensure a rigorous development methodology [18,19]. To overcome the main obstacles during CDSS development and deployment, several of these recommendations suggest focusing on the clinical question to be answered by the model and clinicians’ expectations of the AI-based CDSS in production [17]. Involving health care providers at every stage of the development and implementation process (user-centered design) would be a solution to overcome the applicability barriers [20,21]. This study is therefore an application of these frameworks, proposing a predevelopment survey before the creation of an AI-based CDSS in the ICU.

In France, the decision to initiate, adjust settings, and discontinue dialysis, including CRRT, are made exclusively by intensivists, without systematic involvement of nephrologists. National guidelines provide general recommendations for dialysis management but lack standardized protocols for CRRT weaning [22]. Local protocols may be used in some ICUs, but there is no universal codification of weaning practices in France. This variability further underscores the potential value of a CDSS to support intensivists in this complex task. Our team is currently planning to develop an AI-based CDSS for successful weaning from CRRT in the ICU, defined as no reintroduction of renal assistance within seven days of its interruption [14].

The aim of this study was to survey ICU physicians, before the development of a machine learning predictive model for successful weaning from CRRT, to understand their expectations regarding the proposed tool, their opinion of the chosen clinical problem and their level of knowledge, appease and distrust of AI-based CDSS in general. In particular, we sought to gather insights from physicians working in both university and nonuniversity hospital settings, as we hypothesized that their exposure to technological innovations and clinical workflows might differ.

## Methods

### Study Protocol

A prospective cross-sectional survey study was conducted in France using a declarative survey from December 1, 2023 to April 30, 2024. The survey was hosted on a health data platform using Goupile software [23], and the link was shared to intensive care physicians via the newsletters and social media of the French Society of Anesthesia and Intensive Care (Société Française d’Anesthésie,-Réanimation), the French Intensive Care Society (Société de Réanimation de langue Française), the French College of Nonacademic Hospitals (Collège de Réanimation des hôpitaux Extra-Universitaires de France) and the Association of Private Sector Intensive Care Physicians (Association des Réanimateurs du Secteur Privé). No specific approach was taken to prevent multiple participation in the survey. This study was reported in accordance with the consensus-based Checklist for Reporting of Survey Studies (CROSS) checklist (Multimedia Appendix 1) [24].

### Ethical Considerations

The study was approved by the Ethics and Evaluation Committee for Noninterventional Research of Rouen University Hospital (registration number E2023-61). Participants responded voluntarily after being informed of the purpose of the survey and consenting to the use of their answers. Participation was anonymous and no effort was made to identify individual participants.

### Target Population

The target population included all physicians with clinical activity in intensive care, regardless of their medical specialty, including attending physicians, fellows and junior doctors (final-year residents who have completed their medical thesis and are practicing autonomously under guided supervision according to the French medical education). Participants were not necessarily experts in the field of AI or data analysis and did not receive any additional information on the project beyond what was stated in the questionnaire preamble.

### Survey Instrument

There is currently no standardized questionnaire for AI-based CDSS predevelopment. This questionnaire was developed by 2 intensive care physicians and a medical informatics expert after consultation with experts in data science and user experience. Several meetings were held to ensure that the questions covered relevant and well-established topics in the field of CRRT and AI-based decision support systems, relying on similar studies from the literature. This iterative process allowed to incorporate feedback and adjusted the questions accordingly, although it was not a formal Delphi process. It was developed from instruments already used in the literature to cover all the main axes of reflection relating to the development of a machine learning-based CDSS [20,25-29]. From this literature review and discussions, we identified 4 main axes that would comprehensively capture the necessary information for the future development and implementation of our CDSS. These 4 axes were developed based on their relevance to the successful deployment of AI-based CDSS in clinical practice (Textbox 1).

Textbox 1. **Overview of the key axes explored in the questionnaire.**

- Key drivers (Q1-5): This axis focused on the current attitudes and behaviors regarding decision-making and the specific issue of weaning from CRRT. Understanding these factors is crucial for developing a tool that aligns with clinicians’ decision-making processes and addresses their primary concerns.
- Acceptability (Q6-10): This axis evaluated the current opinions of ICU physicians on AI-based CDSS. Assessing acceptability helps determine the likelihood of adoption and highlights potential barriers that need to be addressed.
- Implementation (Q11-15): This axis explored the critical aspects of using a potential AI-based CDSS, including preferred methods of integration, interface design, and alert thresholds. Insights from this axis are essential for ensuring that the CDSS can be smoothly incorporated into the clinical workflow.
- Usability (Q16-20): This axis examined the real-life operation of the potential AI-based CDSS and the willingness of physicians to adopt it in their everyday practice. Usability is a critical factor in the long-term success of the CDSS, influencing its sustained use and impact on patient care.

The questionnaire consisted of 20 questions on the topic being studied and 6 demographic questions. The 20 questions consisted of 12 statements, 4 multiple-choices, 3 opens and 1 hierarchical question. Statements were answered on a 5-point Likert scale ranging from 1 (strongly disagree) to 5 (totally agree).

The questions also considered acceptance concepts defined by the Unified Theory of Acceptance and Use of Technology (UTAUT) model [30]. In the context of technology, acceptance was defined as the willingness, intention and internal motivation to use a technology as a result of positive attitudes toward the technology or system [31]. The UTAUT model comprised four main concepts: performance expectancy (user’s confidence that using technology will benefit his work performance), effort expectancy (user’s beliefs of how easy it is to use the system), social influences (how much the user feels that significant others believe that they should use the technology) and facilitating conditions (user’s beliefs on organizational and technical support to use the system).

As a first step, the questionnaire was pretested by a panel of 4 intensive care physicians who were not experts in the field of AI or CDSS. Each participant answered the questions and assessed their comprehensibility on a 5-points Likert scale, suggesting modifications if necessary. The proposed modifications were considered, questions with comprehension problems were reformulated and the questionnaire was returned to the panel for final approval. The finalized version of the questionnaire was sent to survey participants. The detailed questionnaire with the rationale for each question can be found, both in the original French version and translated into English, in MultimediaAppendices 2  3.

### Statistical Analyses

Values are presented as numbers and percentages (n, %) for qualitative variables, and as median and IQR for quantitative variables. On the web questionnaire, the response to each question was mandatory, in order to obtain a low rate of missing data. Statistical analyses were performed in complete case analysis on fully completed questionnaires [32]. Subgroup analyses were performed, comparing physicians from university and nonuniversity hospitals and comparing senior (attending physicians) and junior physicians (including fellows and junior doctors). It was based on an a priori hypothesis that differences in AI adoption might exist between university and nonuniversity centers, as well as across age categories. University hospitals were hypothesized to have greater exposure to technological innovations, potentially influencing adoption rates. Similarly, younger physicians were assumed to be more familiar with and open to AI technologies, given their higher exposure to digital tools during training. Bivariate analyses were performed using Wilcoxon rank sum tests for quantitative variables, and *χ*^2^ or Fisher exact tests for qualitative variables.

To control for potential confounding factors, multivariate analyses were performed using ordinal logistic regression models. The variables included in the models were selected based on their clinical relevance and their potential impact on the outcomes of interest. These variables included age, years of ICU experience, medical specialty and workplace setting (university or nonuniversity hospital). The dependent variables corresponded to specific survey questions, allowing us to evaluate the independent effect of each factor on the outcomes while accounting for confounding.

All tests were 2-tailed and interpreted at *P*<.05 significance threshold. Statistical analyses were performed using the R software version 4.1.3 (R Foundation for Statistical Computing).

## Results

### Respondent Characteristics

A total of 171 complete responses were received during the study period. It is important to note that the precise number of ICU physicians to whom the survey was delivered was not exhaustively documented. The survey was distributed through the newsletters of French intensive care societies and associations, as well as their social media platforms. While the total number of senior physicians working in the ICU in France is challenging to ascertain, an estimate based on a report from the College of Intensive Care Medicine Teachers (Collège des Enseignants de Médecine Intensive Réanimation) in 2021 approximated this figure to be around 2350 senior physicians [33]. Our study also included junior doctors who are not counted in this figure. There are approximately 500 junior doctors training in ICUs annually in France, which means the actual denominator for the survey population would be closer to 2850. Hence, the estimated response rate for the survey is approximately 6.0% (168/2850, considering that 2 respondents were from Belgium and 1 from Switzerland).

The total group had a median age of 39 (IQR 33‐45) years and a median of 9 (IQR 3‐14) years of ICU work experience (Table 1). Out of the 171 physicians, most of them had a training in anesthesiology and intensive care (109, 63.7%) and worked in university hospitals (102, 59.6%). The median age of physicians surveyed in university centers was younger (36 years [IQR 32‐43] versus 42 [IQR 36‐50]; *P*<.001), with fewer years of experience (5 years [IQR 2‐12] versus 11 [IQR 6‐16]; *P*<.001), and more from the anesthesia and intensive care training (81/102, 79.4% vs 28/69, 40.6%; *P*<.001).

**Table 1.** Characteristics of survey respondents.

Characteristics	Overall(n=171)	Nonuniversity hospitals(n=69)	University hospitals(n=102)	*P* values
Age, years, median (IQR)^a^	39 (33‐45)	42 (36‐50)	36 (32‐43)	<.001
Years of experience in the ICU^b^, median (IQR)	9 (3-14)	11 (6‐16)	5 (2-12)	<.001
Status, n (%)				.05
Attending	138 (81)	62 (90)	76 (75)	
Fellow	21 (12)	5 (7.2)	16 (16)	
Junior doctor	12 (7)	2 (2.9)	10 (9.8)	
Medical specialty, n (%)				<.001
Anesthesiology and intensive care	109 (64)	28 (41)	81 (79)	
Intensive care medicine	56 (33)	37 (54)	19 (19)	
Emergency medicine	3 (1.8)	2 (2.9)	1 (1.0)	
Cardiology	1 (0.6)	1 (1.4)	0 (0)	
Nephrology	1 (0.6)	0 (0)	1 (1.0)	
Pneumology	1 (0.6)	1 (1.4)	0 (0)	
Hospital type, n (%)				<.001
University hospital	102 (60)	0 (0)	102 (100)	
General hospital	58 (34)	58 (84)	0 (0)	
Private hospital	7 (4.1)	7 (10)	0 (0)	
Nonprofit private hospital	3 (1.8)	3 (4.3)	0 (0)	
Military hospital	1 (0.6)	1 (1.4)	0 (0)	
Country, n (%)				.99
Belgium	2 (1.2)	1 (1.4)	1 (1.0)	
France	168 (98)	68 (99)	100 (98)	
Switzerland	1 (0.6)	0 (0)	1 (1.0)	

a IQR: interquartile range.

b ICU: intensive care unit.

### Overview of the Problem and Current Practices (Key Drivers)

**Figure 1.**
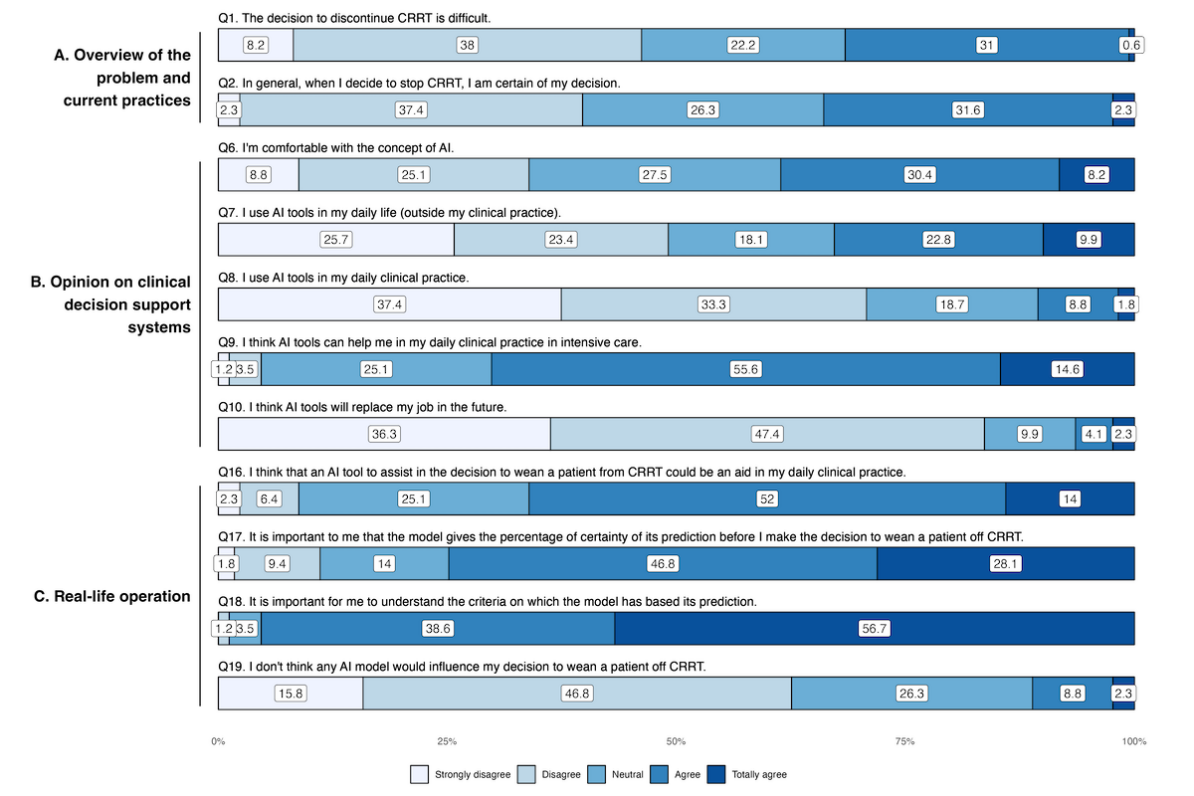
Clinicians’ answers to statements questions regarding (A) the overview of the problem and current practices, (B) opinion on clinical decision support systems, and (C) real-life operation, willingness to adopt in everyday practice. Results are presented as percentages. AI: artificial intelligence; CRRT: continuous renal replacement therapy.

Concerning the CRRT-weaning decision, doctors disagreed on the difficulty of decision-making, with 46.2% (79/171) disagreeing or strongly disagreeing with the weaning difficulty proposition (Q1) and 31.6% (54/171) agreeing or totally agreeing (Figure 1A and Table 2). Regarding the certainty of the weaning decision (Q2), responses were mixed, with 33.9% (58/171) of doctors agreeing or totally agreeing with the proposition and 39.7% (68/171) disagreeing or strongly disagreeing. There was no difference between academic and nonacademic physicians (Table 2 and Figure S1 in Multimedia Appendix 4), and between different levels of training (Table S1 and Figure S2 in Multimedia Appendix 4). Physicians had a good idea of the CRRT weaning failure rate (Q3), with a median rate estimated at 33% (IQR 25‐50), which is close to the rates of 35% to 54% reported in the literature [34,35].

**Table 2.** Responses to statements regarding the overview of the problem and the opinion on clinical decision support systems on a Likert scale from 1 (strongly disagree) to 5 (totally agree). Results are presented as median (IQR).

Question	Overall(N=171)	Nonuniversity hospitals(n=69)	University hospitals(n=102)	*P* values	Adjusted *P* values^a^
Overview of the problem and current practices					
Q1: The decision to discontinue CRRT^b^ is difficult.	3 (2-4)	2 (2-4)	3 (2-4)	.30	.12
Q2: In general, when I decide to stop a CRRT, I am certain of my decision.	3 (2-4)	3 (2-4)	3 (2-4)	.60	.57
Opinion on clinical decision support systems					
Q6: I am comfortable with the concept of artificial intelligence.	3 (2-4)	3 (2-4)	3 (2-4)	.11	.72
Q7: I use AI^c^ tools in my daily life (outside my clinical practice).	3 (1-4)	2 (1-4)	3 (2-4)	.22	.06
Q8: I use AI tools in my daily clinical practice.	2 (1-3)	2 (1-2)	2 (1-3)	.07	.80
Q9: I think AI tools can help me in my daily clinical practice in intensive care.	4 (3-4)	4 (3-4)	4 (4-4)	.08	.41
Q10: I think AI tools will replace my job in the future.	2 (1-2)	2 (1-2)	2 (1-2)	.50	.18

a Adjustment on age, years of experience and medical specialty.

b CRRT: continuous renal replacement therapy.

c AI: artificial intelligence.

**Figure 2.**
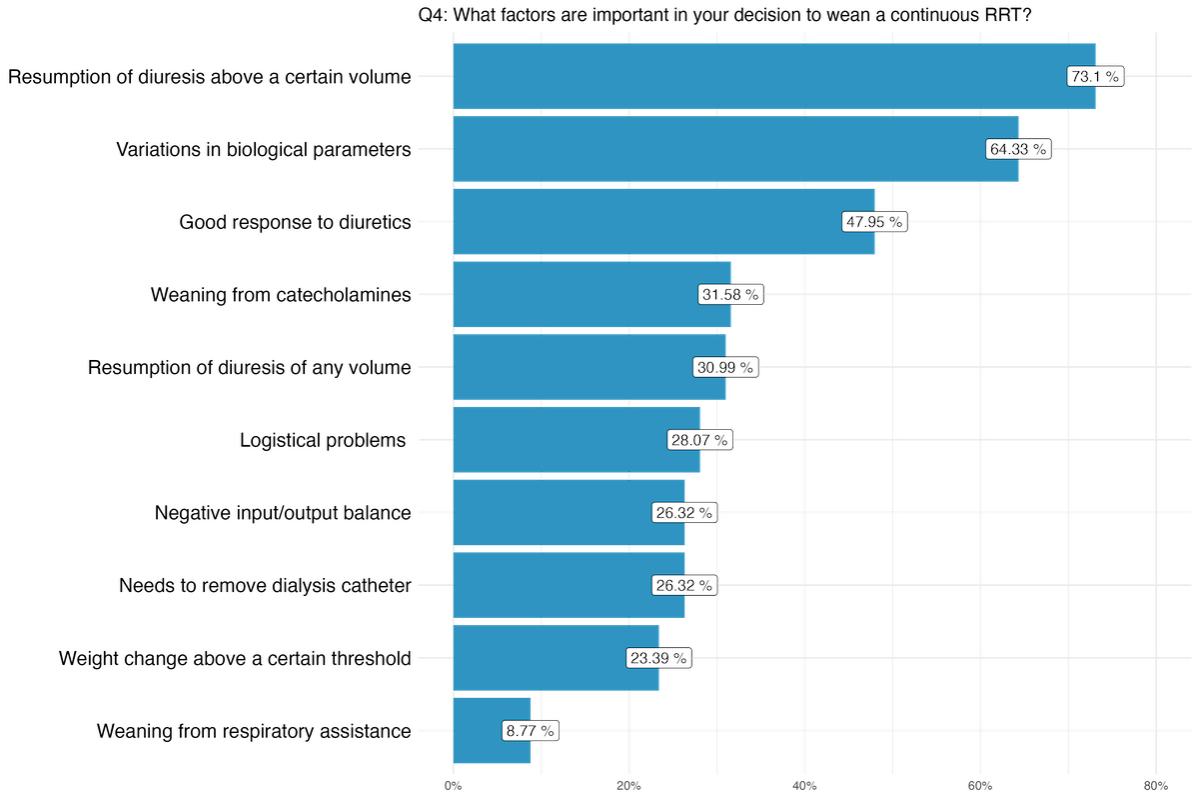
Variables relevant to the clinical decision to wean from continuous renal replacement therapy (percentages of respondents choosing each variable). RRT: renal replacement therapy.

Regarding the variables affecting the decision to wean from CRRT (Q4), respondents mainly chose the resumption of diuresis above a certain volume (125/171, 73.1%), variation in biological settings (110/171, 63.3%) and good response to diuretics (82/171, 47.9%; Figure 2). They were then asked to rank these variables in order of importance from 1 the most important, to 10, the least important. The most important variable for the respondents was the resumption of diuresis above a certain volume, with 33.9% (58/121) of respondents ranking it in first place and 11.7% (20/121) ranking it second. Other variables frequently ranked highly included variations in biological parameters, good response to diuretics, and weaning from catecholamines.

### Opinion on Clinical Decision Support Systems (Acceptability)

Respondents did not seem particularly comfortable with the concept of AI (66/171, 38.6% agreed or totally agreed with Q6) and did not seem to use AI tools in their daily lives (84/171, 49.1% disagreed or strongly disagreed with Q7) and even less so in their clinical activity with 70.7% (121/171) disagreeing or strongly disagreeing with Q8 (Figure 1B and Table 2). Junior physicians made greater use of AI tools in their daily lives, with 45.5% (15/33) agreeing or totally agreeing with Q7 compared to 29.7% (41/138) of senior doctors (*P*=.03; Table S1 in Multimedia Appendix 4). However, the surveyed physicians were very favorable to the use of AI tools to help them in their clinical practice, with 70.2% (120/171) agreeing or totally agreeing with Q9, with a priori no fear, since 83.7% (143/171) of respondents disagreeing or totally disagreeing with Q10 concerning the fact that AI could replace their job in the future. This enthusiasm seemed consistent between the different levels of training (94/138, 68.1% agreed or totally agreed vs 26/33, 78.8%; *P*=.08) and the university hospitals status (78/102, 76.5% agreed or totally agreed vs 42/69, 60.9%; *P*=.41; Figure S1 in Multimedia Appendix 4).

### Implementation in Daily Clinical Practice

**Figure 3.**
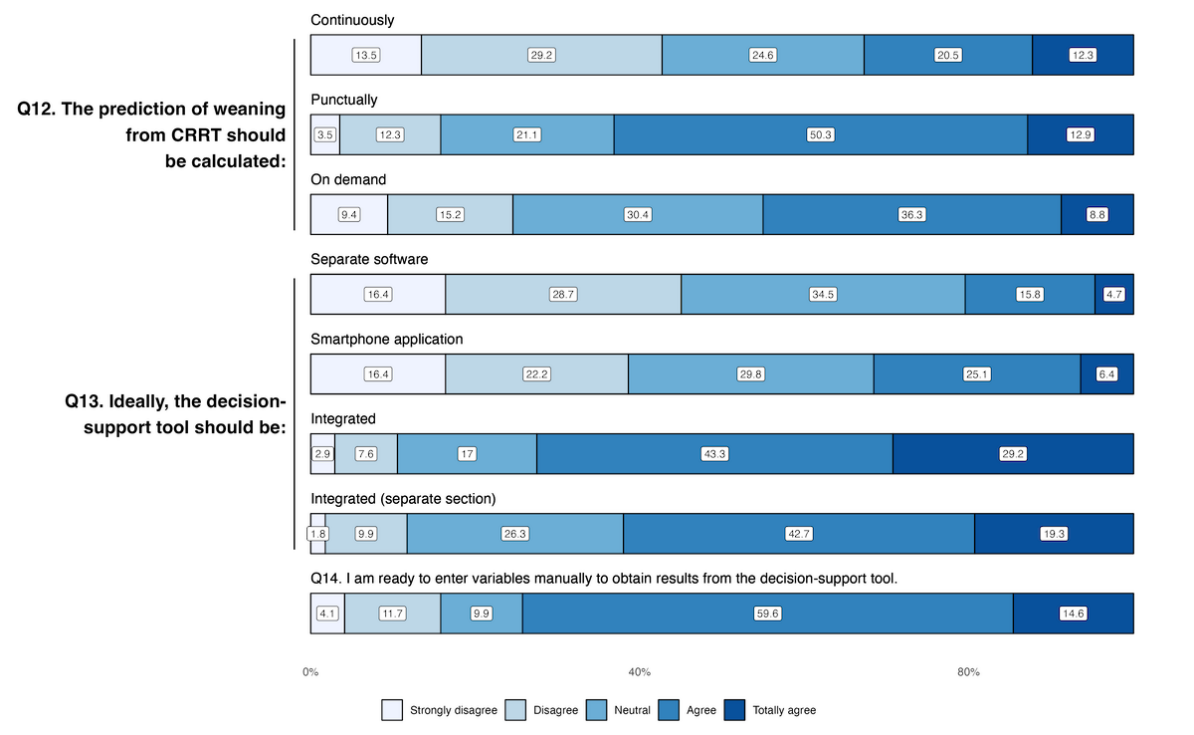
Answers to statements questions regarding the implementation of the clinical decision support system in daily clinical practice. AI: artificial intelligence; CRRT: continuous renal replacement therapy.

When asked about the preferred timing for computing the prediction of CRRT weaning (Q12), respondents preferred it to be rendered punctually at a specific time during the day, for example during the morning round (108/171, 63.2% agreed or totally agreed) versus on demand only (77/171, 45.1% agreed or totally agreed) or continuously (56/171, 42.7% agreed or totally agreed) (Figure 3 and Table 3). Senior physicians were more inclined to choose a prediction at a specific time of day (93/138, 67.4% agreed or totally agreed vs 15/33, 45.5%; *P*=.01), whereas juniors were more interested in an on-demand prediction only (20/33, 60.7% agreed or totally agreed, Table S1 in Multimedia Appendix 4). Regarding the choice of design for the CDSS (Q13), respondents were generally in favor of it being integrated within the ICU’s electronic health record (EHR), whether with the usual monitoring data (124/171, 72.5% agreed or totally agreed) or on a separate section of the software (106/171, 62% agreed or totally agreed). Out of the 171 respondents, 141 (82.5%) physicians declared that their hospital was equipped with an ICU EHR (Q11), with greater computerization in university hospitals (93/101, 91% vs 48/79, 70%; *P*<.001). Most physicians agreed to manually input variables to be added to the model (127/171, 74.2% agreed or totally agreed). The median number of acceptable variables to enter manually was 5 (IQR 4‐6) (Q15).

**Table 3.** Responses to statements regarding the implementation of the clinical decision support system in daily clinical practice on a Likert scale from 1 (strongly disagree) to 5 (totally agree). Results are presented as the median (IQR).

Question	Overall(N=171)	Nonuniversity hospitals(n=69)	University hospitals(n=102)	*P* values	Adjusted *P* values^a^
Q12: The prediction of weaning from CRRT^b^ should be calculated:					
Continuously	3 (2-4)	3 (2-4)	3 (2-4)	.59	.93
Punctually, at a specific time (eg during morning round)	4 (3-4)	4 (3-4)	4 (3-4)	.78	.20
Punctually, on demand only	3 (3-4)	3 (2-4)	3 (3-4)	.50	.90
Q13: Ideally, the decision-support tool should be:					
A separate software application	3 (2-3)	3 (2-3)	3 (2-3)	.20	.97
A smartphone or tablet application	3 (2-4)	3 (2-4)	3 (2-4)	.71	.83
Integrated into the ICU^c^ patient management software and visible at the same time as other vital parameters	4 (3-5)	4 (3-5)	4 (3-5)	.99	.69
Integrated into the ICU patient management software and visible in a separate section (action required to view prediction results)	4 (3-4)	4 (3-4)	4 (3-4)	.11	.70
Q14: I’m ready to enter variables manually to obtain results from the decision- support tool	4 (3-4)	4 (4-4)	4 (3-4)	.20	.42

a Adjustment on age, years of experience, and medical specialty.

b CRRT: continuous renal replacement therapy.

c ICU: intensive care unit.

### Real-Life Operation, Willingness to Adopt in Everyday Practice (Usability)

**Figure 4.**
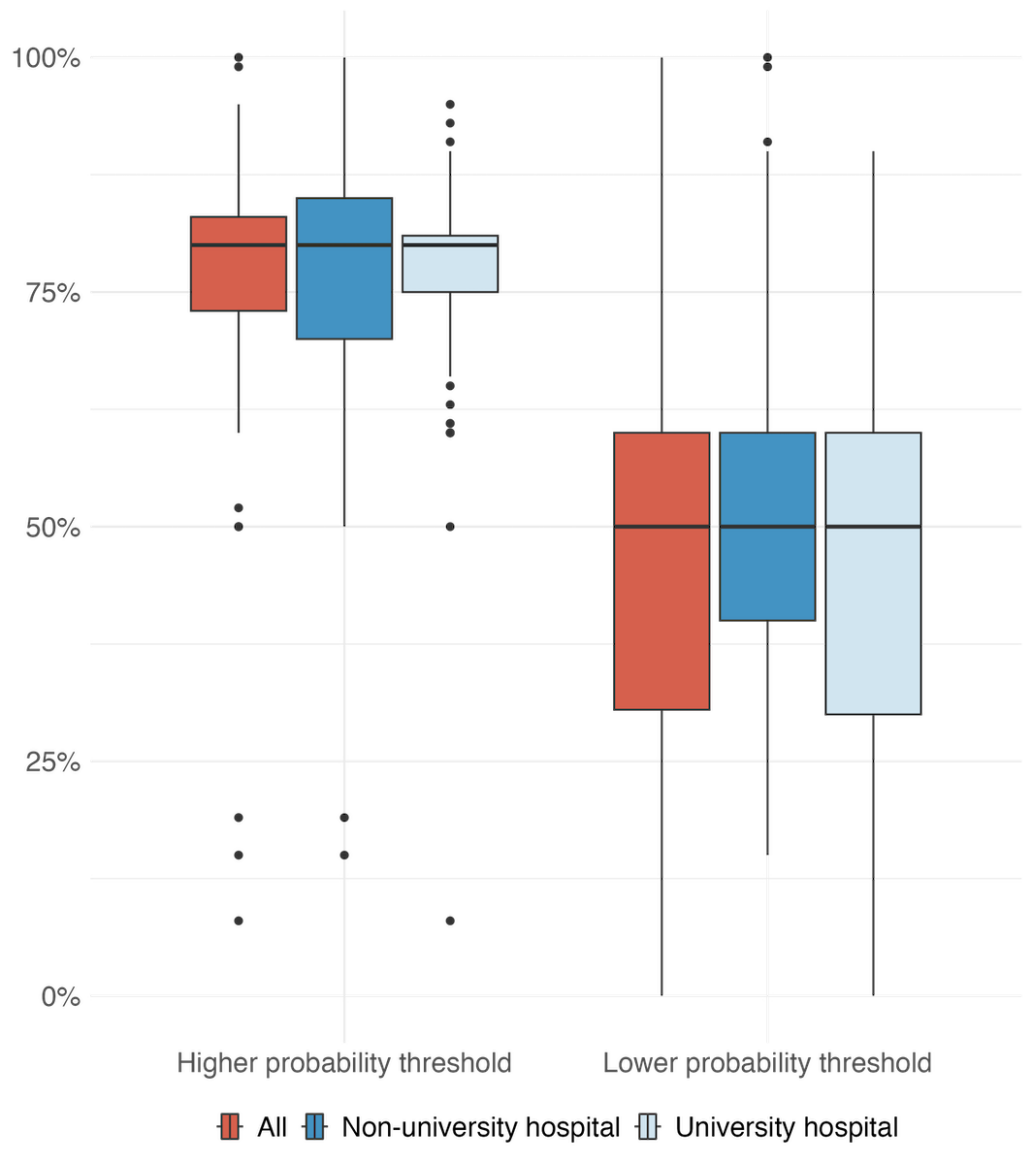
Prediction thresholds described by clinicians as having an impact on their clinical decision-making on continuous renal replacement therapy weaning.

Respondents agreed that an AI-based CDSS to assist in decisions about weaning patients from CRRT could be an aid in daily clinical practice, with 66% (113/171) either agreeing or totally agreeing (Q16). The adherence appeared to be higher in university hospitals (78/102, 76.5% agreeing or totally agreeing) compared to nonuniversity settings (35/69, 50.7%; *P*<.001) (Figure 1C and Table 4). However, after adjusting for confounders such as age, the difference was no longer statistically significant (adjusted *P*=.63), suggesting that the observed difference may be more related to the younger age profile of university hospital respondents. Similarly, younger physicians were more likely to express support for AI tools (27/33, 81.8% agreed or totally agreed vs 86/138, 62.3%; *P*=.01) (Table S1 in Multimedia Appendix 4). The explicability of the model was an important concern, with 74.9% (128/171) of respondents agreeing or totally agreeing that the AI-based CDSS should provide a percentage of certainty with the prediction provided (Q17), and 95.3% (163/171) that it was important to understand the criteria on which the model has based its prediction (Q18). Only 11.1% (19/171) doubted that the model would influence their decision to wean a patient off CRRT (Q19). Finally, interrogated physicians indicated a low probability threshold provided by the model below which they would not consider weaning a patient from CRRT at a median of 50% (IQR 30‐60) and a threshold above which they would consider weaning at 80% (IQR 73‐85) (Figure 4). These thresholds highlight the range within which model predictions could inform clinical decisions and suggest that physicians prefer a cautious approach, favoring higher certainty for weaning decisions while remaining conservative when uncertainty is high.

**Table 4.** Responses to statements regarding the real-life operation and willingness to adopt in everyday practice on likert scale from 1 (strongly disagree) to 5 (totally agree). Results are presented as median (IQR).

Question	Overall(N=171)	Nonuniversity hospitals(n=69)	University hospitals(n=102)	*P* values	Adjusted *P* values^a^
Q16: I think that an AI^b^ tool to assist in the decision to wean a patient from CRRT^c^ could be an aid in my daily clinical practice.	4 (3-4)	4 (3-4)	4 (4-4)	<.001	.63
Q17: It is important to me that the model gives the percentage of certainty of its prediction before I make the decision to wean a patient off CRRT.	4 (4-5)	4 (3-5)	4 (4-5)	.51	.29
Q18: It is important for me to understand the criteria on which the model has based its prediction.	5 (4-5)	5 (4-5)	5 (4-5)	.20	.11
Q19: I don’t think any AI model would influence my decision to wean a patient off CRRT.	2 (2-3)	2 (2-3)	2 (2-3)	.06	.52

a Adjustment on age, years of experience and medical specialty.

b AI: artificial intelligence.

c CRRT: continuous renal replacement therapy.

The responses to the survey have enabled us to draft an initial list of specifications fort the development of the CDSS to assist in weaning from CRRT. These specifications and the consequences for further development are detailed in Table 5.

**Table 5.** Specifications and consequences for development of the clinical decision support system.

Feature	Specification	Consequences for development
Overview of the problem and current practices (key drivers)		
Variables to include	Key variables such as resumption of diuresis, biological markers and response to diuretics.	Ensure these variables are accurately and automatically collected from EHR^a^ to minimize manual input errors.
Implementation in daily clinical practice		
Manual data entry	Willingness to manually enter up to 5 variables.	Automate the collection of as many variables as possible by integrating with ICU^b^ EHR.
Integration with existing systems	Integration with ICU EHR, predictions visible alongside vital parameters.	Develop seamless integration with EHR systems to ensure data accessibility and workflow efficiency.
Timing of predictions	Predictions computed at a specific time, such as morning rounds.	Schedule predictions to align with clinical routines, reducing the need for continuous monitoring.
User interface	User-friendly, minimal additional workload, no system switching.	Design an intuitive interface that fits into clinicians’ workflow to enhance usability.
Real-life operation, willingness to adopt in everyday practice (usability)		
Explainability	Clear explanations of model predictions.	Develop an interface that visually explains the predictions to build trust among users.
Alert thresholds	Probability thresholds below 50% for considering not weaning and above 80% for considering weaning.	Calibrate the model to achieve the desired thresholds for user confidence and actionability.

a EHR: electronic health record.

b ICU: intensive care unit.

## Discussion

### Principal Findings

Our study provides insights into the expectations and attitudes of ICU physicians regarding the use of AI-based CDSS for weaning from CRRT. This predevelopment study is a fundamental step toward a user-centered approach to the development of a decision support tool. The results of our survey suggest that while physicians are not generally comfortable with AI and have limited experience using it, 70.2% (120/171) expressed a favorable view toward integrating AI tools in clinical practice, with no apparent fear of this technology. Moreover, our results suggest a significant interest and perceived necessity among French intensivists for a CDSS tailored to assist in the weaning process from CRRT.

### Attitudes Toward AI and CDSS

Physicians in our study, particularly senior ones, did not seem entirely comfortable with the concept of AI. This highlights the need for comprehensive training and education to improve AI literacy among clinicians, fostering a more favorable environment for the integration of CDSS [17]. How physicians interpret and act on CDSS predictions can substantially impact patient care [36]. Despite this discomfort, there was strong support for AI-based CDSS to aid clinical decision-making, underscoring the potential acceptance of a well-designed CDSS. This positive outlook towards AI is consistent with other studies showing clinicians’ openness to AI-based CDSS if they enhance clinical practice and improve patient outcomes [37]. In a similar study conducted among Dutch ICU physicians in university hospitals found that 86% of respondents believed AI could support them in their work as physicians [25]. In addition, the surveyed physicians showed no significant fear that AI would replace their jobs, with 83.7% (143/171) of respondents disagreed or strongly disagreed with this idea. The relatively young median age of respondents, 39 years (IQR 33‐45) compared to a national median of 43 (IQR 35‐54) in 2021 [33], may also explain the higher overall willingness to adopt AI-based CDSS in clinical practice.

### Preferences for Implementation

Regarding the implementation of a CDSS for CRRT weaning, our survey revealed that physicians preferred a tool that computes the prediction of weaning at a specific time, rather than continuously. This is consistent with previous studies suggesting that continuous monitoring can lead to alarm fatigue and decrease clinician trust in the tool. In a review of 89 articles on CDSS in various medical fields, Jankovic et al [38] showed that poorly performing CDSS with frequent alerts could contribute to clinicians’ frustration and burnout. Furthermore, respondents preferred a CDSS integrated into the ICU patient management software, visible alongside other vital parameters, rather than a new interface. This aligns with previous research showing that integration into clinical workflows is key to improving usability and adoption [39]. A user-centered design is critical, as highlighted by Zikos and DeLellis [40]*,* who identified four real-life “pain points” where AI can add significant value for clinicians, including selecting effective interventions.

### Factors Influencing Weaning Decision

Our study also highlights the importance of understanding the factors that influence physicians’ decisions to wean patients from CRRT. The most frequently cited factors were resumption of diuresis above a certain volume, variation in biological settings, and good response to diuretics. These factors align with a previous systematic review, which identified similar predictors for successful RRT weaning, such as the urine output before RRT discontinuation and biochemical criteria (eg, serum urea, serum creatinine and creatinine clearance) [13]. These variables should therefore be included in the CDSS model to ensure clinical relevance in the weaning predictions. With the CRRT weaning success rate reported between 35% and 54% in the literature [34,35], our CDSS seems well-positioned to support this complex decision-making, as indicated by the responses of the surveyed clinicians.

### Real-Life Operation and Adoption

This survey emphasizes the importance of understanding end-user needs and expectations during the predevelopment phase, which is crucial for successful deployment and adoption in clinical practice [20]. The first step is ensuring that the AI-based CDSS addresses the specific needs of clinicians [41]. Moreover, ensuring the explicability of AI models is crucial to avoid the “black box” effect. Physicians need to understand the factors driving the model’s prediction to trust and use these tools effectively. Transparent AI models with clear insights into their decision-making processes can enhance user acceptance and facilitate smoother integration into clinical practice [42].

### Limitations

It is worth noting that our study has limitations. First, the response rate was low and introduced the possibility of selection bias, as those more interested in AI may have been more likely to respond. This could have overestimated the acceptance or perceived usefulness of AI-based CDSS. As a result, caution is necessary when generalizing our results to the broader population of ICU physicians. In addition, the lack of an exhaustive list of ICU physicians to whom the survey was distributed further complicated the estimation of a precise response rate and the generalizability of the findings. While a significant proportion of respondents reported limited familiarity with AI, suggesting some mitigation of this bias, the possibility remains that the survey sample was not representative of the entire ICU physician population in France. Low response rates are a common challenge in survey-based research targeting healthcare professionals, who often face significant time constraints. To improve future response rates, strategies such as personalized invitations, follow-up reminders, incentives, and more direct communication channels (eg, targeted emails, professional networks) could be considered. Despite these challenges, our sample size was larger than similar studies ranging, from 7 to 93 participants [25-27,29] and included a diverse group of physicians from both university and nonuniversity hospitals, public and private sectors, and various level of training.

Second, our population skewed younger, with a median age of 39 years compared to the national median of 43 years. This could be attributed to the digital distribution of the survey via newsletters and social media platforms, which may have engaged younger physicians more effectively. The overrepresentation of younger intensivists could influence the generalizability of our findings, as younger physicians are often more familiar with technology and may exhibit greater openness toward AI adoption compared to their older counterparts. This positive bias toward AI could have amplified the generally favorable attitudes observed in our survey. Future studies should aim for a more representative sampling across age groups to ensure broader applicability of the results and to capture potentially differing perspectives from older physicians.

Third, we observed an overrepresentation of physicians from university hospitals, with 60% of respondents working in these centers, compared to around 50% according to recent estimates [33]. University hospital physicians may have greater exposure to technological innovations and more formal training in AI. This might make them more inclined to adopt new technologies like AI-based CDSS. This could have contributed to the positive outlook on AI tools in our survey.

Fourth, self-reporting bias could also influence the results, as participants may have provided responses they perceive as desirable. Finally, while our questionnaire was rigorously designed by a multidisciplinary team and based on instruments in similar studies, such as van der Meijden et al [25]*,* there is currently no validated tool for predevelopment surveys of AI-based CDSS. This may affect the reliability and comparability of our results. In addition, there may have been some comprehension issues with certain questions, as some respondents probably inverted values for decision thresholds in Q20. Finally, this survey was conducted in France, targeting a specific audience for our CDSS, and the results may not be generalizable to other countries with different health care systems and practices.

### Future Directions

Based on the survey results, the next steps in developing the CDSS for CRRT weaning will follow the specifications derived from the respondent’s feedback, particularly regarding the selection of variables and the number of variables to incorporate into the model. The integration of the CDSS into existing ICU EHR should be prioritized, as this was preferred by most respondents, to ensure seamless integration into clinical workflows. The probability thresholds identified in the survey (50.0% and 80.0%) could guide the CDSS by defining actionable zones for clinical recommendations, with flexibility for customization based on institutional practices. These thresholds would help translate model predictions into practical decision-making support while preserving clinical discretion. In addition, clear explanations of predictions, including criteria and levels of certainty, must be incorporated to align with the strong preference for model transparency expressed by respondents.

Beyond model development, it is crucial to maintain ongoing user engagement throughout the process. This involves returning to the users with models and interface proposals to gather feedback on the model’s functionality and interpretability. Iterative development, involving continuous user input and feedback, is known to enhance acceptance and integration of AI-based CDSS in practice [43,44]. This approach helps ensure that the final product aligns closely with user expectations and needs, improving usability and satisfaction. After refining the model, a prospective study will evaluate its performance in real-world settings and its ability to generalize across different clinical contexts. The goal is to validate the model not only in controlled environments but also in diverse ICU settings to ensure its broader applicability and effectiveness.

In conclusion, our study provides valuable insights into ICU physicians’ expectations and opinions regarding an AI-based CDSS for CRRT weaning, enabling us to draft an initial set of specifications. While there is cautious optimism about the use of AI in clinical practice, significant efforts are needed to address concerns about usability, integration, and transparency. By understanding and incorporating the needs and preferences of clinicians, we can develop a CDSS that is both effective and widely accepted. This work is a preliminary step in guiding future research continuing to involve health care providers in the development process and promoting user-centered design for the ongoing development of a CDSS to guide CRRT weaning in the ICU. This approach will ultimately enhance the adoption and impact of AI-based CDSS in critical care settings, improving patient outcomes and optimizing resource utilization.

## Supplementary material

10.2196/63709 Multimedia Appendix 1 Consensus-Based Checklist for Reporting of Survey Studies (CROSS) checklist.

10.2196/63709 Multimedia Appendix 2 Questionnaire translated into English with rational for each question.

10.2196/63709 Multimedia Appendix 3 Questionnaire in its original French version.

10.2196/63709 Multimedia Appendix 4 Responses to statements on a Likert scale from 1 (strongly disagree) to 5 (totally agree) according to level of training.

## Abbreviations

AI: artificial intelligence
AKI: acute kidney injury
CDSS: clinical decision support systems
CRRT: continuous renal replacement therapy
EHR: electronic health record
ICU: intensive care unit
IQR: interquartile range
ML: machine learning
RRT: renal replacement therapy
UTAUT: unified theory of acceptance and use of technology
