# Supplementary material for: Expectations of Intensive Care Physicians Regarding an AI-Based Decision Support System for Weaning From Continuous Renal Replacement Therapy: Predevelopment Survey Study
Source: JMIR Med Inform. 2025 Apr 23;13:e63709. doi: 10.2196/63709 (PMC12043247; doi:10.2196/63709)
Supplement: Multimedia Appendix 2 [file medinform-v13-e63709-s002.pdf]

MULTIMEDIA APPENDIX 2

Expectations of intensive care physicians regarding a decision support tool for weaning from continuous renal replacement therapy: a pre-development survey study

Questionnaire translated into English with rational for each question

This questionnaire is part of the development of a decision support model for weaning patients admitted to intensive care from continuous renal replacement therapy, based on artificial intelligence technologies. Weaning is defined as the cessation of all forms of renal replacement therapy within 7 days, without the need for restarting the therapy.
Numerous recent studies have published decision-support algorithms, but few have made it past the stage of real-life experimentation. These implementation problems, i.e. the transition from a theoretical algorithm to an application in current clinical practice, may be due to insufficient preparation upstream of algorithm development.
The aim of this pre-development study is 1) to understand clinicians' current decision-making habits regarding weaning from continuous renal replacement therapy in the ICU and 2) to assess users' opinions, willingness to adopt and expectations prior to development.
By voluntarily answering this questionnaire, you consent to the use of your answers for scientific research purposes.

General information

- Type of hospital
- Specialty: anesthesia-intensive care, medical intensive care, other
- Status
- Years of senior practice in intensive care
- Age
- Country of practice

Overview of the problem and practices (key drivers)

**Question 1:** The decision to discontinue continuous renal replacement therapy (RRT) is difficult.<br>**Response:** Likert (Strongly disagree, Disagree, Neutral, Agree, Totally agree)<br>**Rationale:** To understand whether the decision to stop RRT is perceived as clinically complex and whether a decision support tool is relevant in this context.
**Question 2:** In general, when I decide to stop a continuous RRT, I am certain of my decision.<br>**Response:** Likert (Strongly disagree, Disagree, Neutral, Agree, Totally agree)<br>**Rationale:** To understand whether the decision to stop RRT is perceived as clinically complex and whether a decision support tool is relevant in this context.
**Question 3:** In your opinion, what is the percentage of failed weaning from continuous RRT (restarting RRT within 7 days)?<br>**Response:** Integer (0 to 100)<br>**Rationale:** Determine the level of uncertainty and perceived prevalence of RRT weaning failure.
**Question 4:** What factors are important in your decision to wean a continuous RRT?<br>**Response:** multiple choice<br>● Resumption of diuresis above a certain volume<br>● Resumption of diuresis of any volume<br>● Good response to diuretics

- Weaning from catecholamines<br>- Weaning from respiratory assistance<br>- Weight change above a certain threshold<br>- Negative input/output balance<br>- Variations in biological parameters (plasma/urinary)<br>- Need to remove dialysis catheter (infection, thrombosis, dysfunction, etc.)<br>- Logistical problems (circuit coagulation, machine availability, nurse availability)<br>- Other (free text)<br>**Rationale:** Understand the medical decision-making process and list the factors that influence it.
**Question 5:** Of the 10 preceding factors influencing your decision to wean off continuous RRT, rank them from 1 (most important) to 10 (least important).<br>**Response:** Ranking<br>**Rationale:** Identify important variables to incorporate into an AI clinical decision support system.

Opinion on clinical decision support systems (acceptability)

**Question 6:** I'm comfortable with the concept of artificial intelligence (AI).<br>**Response:** Likert (Strongly disagree, Disagree, Neutral, Agree, Totally agree)<br>**Rationale:** Determine the level of familiarity with AI tools to dichotomize expert and novice users.<br>**Concept**: experience
**Question 7:** I use AI tools in my daily life (outside my clinical practice).<br>**Response:** Likert (Strongly disagree, Disagree, Neutral, Agree, Totally agree)<br>**Rationale:** Determine level of familiarity with AI tools in general.<br>**Concept**: experience
**Question 8:** I use AI tools in my daily clinical practice.<br>**Response:** Likert (Strongly disagree, Disagree, Neutral, Agree, Totally agree)<br>**Rationale:** Determine the level of familiarity with AI tools in the care setting.<br>**Concept**: experience
**Question 9:** I think AI tools can help me in my daily clinical practice in intensive care.<br>**Response:** Likert (Strongly disagree, Disagree, Neutral, Agree, Totally agree)<br>**Rationale:** Determining the willingness to implement AI models in routine clinical practice.<br>**Concept**: voluntariness of use
**Question 10:** I think AI tools will replace my job in the future.<br>**Response:** Likert (Strongly disagree, Disagree, Neutral, Agree, Totally agree)<br>**Rationale:** Determining the perceived professional threat posed by AI tools.<br>**Concept**: experience

Usage, interface, warning thresholds (implementation in daily clinical practice)

Let's imagine a decision-support tool that predicts continuous RRT weaning (i.e. cessation without the need for retreatment within 7 days).

**Question 11:** My department is equipped with software for managing intensive care patients (monitoring vital parameters, prescriptions, etc.).<br>**Response:** Binary (yes/no)<br>**Rationale:** Computerization inventory to determine implementation potential with existing software. Determine whether the user uses a computerized tool in his daily practice.

**Question 12:** The prediction of weaning from continuous RRT should be calculated:<br>**Response:** Likert (Strongly disagree, Disagree, Neutral, Agree, Totally agree) for each proposition:<br>● Continuously<br>● Punctually, at a specific time (e.g. during morning round)<br>● Punctually, on demand only<br>**Rationale:** Determine at what point in the day-to-day management process the tool should be updated.<br>**Concept**: effort expectancy
**Question 13:** Ideally, the decision-support tool should be:<br>**Response:** Likert (Strongly disagree, Disagree, Neutral, Agree, Totally agree) for each proposition:<br>● A separate software application<br>● A smartphone/tablet application<br>● Integrated into the ICU patient management software and visible at the same time as other vital parameters<br>● Integrated into the ICU patient management software and visible in a separate section (action required to view prediction results)<br>● Other (free text)<br>**Rationale:** Understand users' preferences for implementation within existing tools.<br>**Concept**: effort expectancy
**Question 14:** I'm ready to enter variables manually to obtain results from the decision-support tool<br>**Response:** Likert (Strongly disagree, Disagree, Neutral, Agree, Totally agree)<br>**Rationale:** Understanding the demand for model interaction and ease of use.<br>**Concept**: effort expectancy
**Question 15:** Ideally, if data is to be entered manually, what is the maximum number of variables would you be willing to enter?<br>**Response:** Integer<br>**Rationale:** Understand users' desire for parsimony in the model. Number of variables to be entered if a tool external to the patient management software is used.<br>**Concept**: effort expectancy

Real-life operation, willingness to adopt in everyday practice (usability)

**Question 16:** I think that an AI tool to assist in the decision to wean a patient from continuous RRT could be an aid in my daily clinical practice.<br>**Response:** Likert (Strongly disagree, Disagree, Neutral, Agree, Totally agree)<br>**Rationale:** Determine willingness to adopt the model in clinical decision-making.<br>**Concept**: voluntariness of use
**Question 17:** It is important to me that the model gives the percentage of certainty of its prediction before I make the decision to wean a patient off continuous RRT.<br>**Response:** Likert (Strongly disagree, Disagree, Neutral, Agree, Totally agree)<br>**Rationale:** Determining the importance of model explainability in its use.<br>**Concept**: explainability
**Question 18:** It is important for me to understand the criteria on which the model has based its prediction.<br>**Response:** Likert (Strongly disagree, Disagree, Neutral, Agree, Totally agree)<br>**Rationale:** Determining the importance of model explainability in its use.<br>**Concept**: explainability

**Question 19:** I don't think any AI model would influence my decision to wean a patient off continuous RRT.<br>**Response:** Likert (Strongly disagree, Disagree, Neutral, Agree, Totally agree)<br>**Rationale:** Determine whether users do not wish to be influenced in their decision-making by an AI tool.<br>**Concept**: voluntariness of use
**Question 20a:** What would be the probability threshold (in %) given by the model above which I would consider weaning a patient from RRT? (e.g. I would consider weaning RRT if the algorithm predicts weanability with a probability greater than x%).<br>**Question 20b:** What would be the probability threshold (in %) given by the model below which I would not consider weaning a patient from an RRT? (e.g. I wouldn't consider weaning RRT if the algorithm predicts weanability with a probability of less than x%).<br>**Response:** Integer (0-100%)<br>**Rationale:** Determine values that can influence decision-making. Enable model calibration.
