# Supplementary material for: Expectations of Intensive Care Physicians Regarding an AI-Based Decision Support System for Weaning From Continuous Renal Replacement Therapy: Predevelopment Survey Study
Source: JMIR Med Inform. 2025 Apr 23;13:e63709. doi: 10.2196/63709 (PMC12043247; doi:10.2196/63709)
Supplement: Multimedia Appendix 3 [file medinform-v13-e63709-s003.pdf]

MULTIMEDIA APPENDIX 3

Expectations of intensive care physicians regarding a decision support tool for weaning from continuous renal replacement therapy: a pre-development survey study

Questionnaire in its original French version.

Ce questionnaire s'inscrit dans le cadre du développement d'un modèle d'aide à la décision du sevrage de l'épuration extra-rénale continue chez des patients admis en réanimation, basé sur des technologies d'intelligence artificielle. Le sevrage est défini comme un arrêt sans nécessité de reprise d'une épuration extra-rénale, quelle que soit sa modalité, dans les 7 jours. De nombreuses études récentes ont publié des algorithmes d'aide à la décision, cependant peu ont réussies à franchir l'étape de l'expérimentation en vie réelle. Ces problèmes d'implémentation, c'est-à-dire le passage d'un algorithme théorique à une application en pratique clinique courante, peuvent être le fait d'une insuffisante préparation en amont du développement de l'algorithme.
L'objectif de cette étude de pré-développement est de 1) comprendre les habitudes actuelles de prise de décision concernant le sevrage de l'épuration extra-rénale continue en réanimation par les cliniciens et 2) d'évaluer en amont du développement l'opinion, la volonté d'adoption et les attentes des utilisateurs.
En répondant volontairement à ce questionnaire vous consentez à l'utilisation de vos réponses à des fins de recherche scientifique.

Informations générales

- Hôpital d'exercice : CHU, CHG/CH, Établissement de santé privé d'intérêt collectif (ESPIC), Hôpital privé à but lucratif, HIA
- Spécialité : anesthésie-réanimation, MIR, autre
- Statut : MCU/PU, PH/PHc, assistant/AHU/CCA/PHU, docteur junior, autre
- Années de pratique en tant que sénior en réanimation
- Âge
- Pays d'exercice

Etat des lieux du problème et des pratiques

Q1 : La décision d'arrêter une épuration extra-rénale (EER) continue est difficile (Likert : Pas du tout d'accord, Pas d'accord, Neutre, D'accord, Totalement d'accord)
Q2 : En général, quand je décide d'arrêter une EER continue, je suis certain de ma décision (Likert : Pas du tout d'accord, Pas d'accord, Neutre, D'accord, Totalement d'accord)
Q3 : A votre avis quel est le pourcentage d'échec de sevrage de l'EER continue (reprise d'une EER dans les 7 jours) ? (nombre)
Q4 : Quels facteurs sont importants dans votre prise de décision de sevrer une EER continue ? (choix multiples) :

- Reprise d'une diurèse supérieure à un certain volume
- Reprise d'une diurèse, peu importe le volume
- Bonne réponse aux diurétiques
- Sevrage des catécholamines
- Sevrage de l'assistance respiratoire

- Variation du poids au delà d’un certain seuil
- Bilan entrées/sorties négatif
- Variation de paramètres biologiques (plasmatique/urinaire)
- Nécessité de retirer le cathéter de dialyse (infection, thrombose, dysfonction, …)
- Problèmes logistiques (coagulation du circuit, disponibilité des machines d’EER, disponibilité IDE)
- Autre (texte libre)

Q5 : Parmi les 10 facteurs précédents influençant votre décision de sevrer une EER continue, hiérarchisez les de 1 (le plus important) à 10 (le moins important).

Opinion sur les systèmes d'aide à la décision (acceptabilité)

Q6 : Je suis à l'aise avec le concept d'intelligence artificielle (IA) (Likert : Pas du tout d'accord, Pas d'accord, Neutre, D'accord, Totalement d'accord)
Q7 : J'utilise des outils d'IA dans ma vie quotidienne (en dehors de ma pratique clinique) (Likert : Pas du tout d'accord, Pas d'accord, Neutre, D'accord, Totalement d'accord)
Q8 : J'utilise des outils d'IA dans ma pratique clinique quotidienne (Likert : Pas du tout d'accord, Pas d'accord, Neutre, D'accord, Totalement d'accord)
Q9 : Je pense que les outils d'IA peuvent m'aider dans ma pratique clinique quotidienne en réanimation (Likert : Pas du tout d'accord, Pas d'accord, Neutre, D'accord, Totalement d'accord)
Q10 : Je pense que les outils d'IA vont remplacer mon métier dans le futur (Likert : Pas du tout d'accord, Pas d'accord, Neutre, D'accord, Totalement d'accord)

Modalités d'utilisation, interface, seuils d'alerte (implémentation dans la pratique clinique quotidienne)

Imaginons un outil d’aide à la décision qui permette de prédire le sevrage (i.e. arrêt sans nécessité de reprise de l’EER dans les 7 jours) d’un patient sous EER continue en réanimation.
Q11 : Mon service est doté d'un logiciel de gestion de patients de réanimation (surveillance des paramètres vitaux, prescription, etc.) (oui/non)
Q12 : La prédiction du sevrage de l'EER continue devrait être calculée :

- En continue (Likert : Pas du tout d'accord/Pas d'accord/Neutre/D'accord/Totalement d'accord)
- Ponctuellement, à un moment précis (ex: le matin pendant la visite par exemple) (Likert : Pas du tout d'accord/Pas d'accord/Neutre/D'accord/Totalement d'accord)
- Ponctuellement, à la demande uniquement (Likert : Pas du tout d'accord/Pas d'accord/Neutre/D'accord/Totalement d'accord)

Q13 : L’outil d’aide à la décision devrait idéalement être (likert/modalité) :

- Un logiciel à part (Likert : Pas du tout d'accord, Pas d'accord, Neutre, D'accord, Totalement d'accord)
- Une application smartphone/tablette (Likert : Pas du tout d'accord, Pas d'accord, Neutre, D'accord, Totalement d'accord)
- Intégré au logiciel de gestion de patients de réanimation et visible en même temps que les autres paramètres vitaux (Likert : Pas du tout d'accord, Pas d'accord, Neutre, D'accord, Totalement d'accord)
- Intégré au logiciel de gestion de patients de réanimation et visible dans une section à part (action nécessaire pour visualiser les résultats de prédiction) (Likert : Pas du tout d'accord, Pas d'accord, Neutre, D'accord, Totalement d'accord)

- Autre (texte libre)

Q14 : Je suis prêt à saisir des variables manuellement pour obtenir des résultats de l'outil d'aide à la décision ? (oui/non)
Q15 : Idéalement, si les données doivent être entrées manuellement, quel est le nombre maximal de variables que vous accepteriez de saisir ? (nombre)

Fonctionnement en vie réelle, volonté d'adoption en pratique courante (utilisabilité)
Q16 : Je pense qu'un outil d'IA d'aide à la décision de sevrage d'une EER continue pourrait être une aide dans ma pratique clinique quotidienne (Likert : Pas du tout d'accord, Pas d'accord, Neutre, D'accord, Totalement d'accord)
Q17 : Il est important pour moi que le modèle donne le pourcentage de certitude de sa prédiction avant que je prenne la décision de sevrer une EER continue (Likert : Pas du tout d'accord, Pas d'accord, Neutre, D'accord, Totalement d'accord)
Q18 : Il est important pour moi de comprendre sur quels critères le modèle aurait basé sa prédiction (Likert : Pas du tout d'accord, Pas d'accord, Neutre, D'accord, Totalement d'accord)
Q19 : Je pense qu'aucun modèle d'IA n'influencerait ma décision de sevrer une EER continue (Likert : Pas du tout d'accord, Pas d'accord, Neutre, D'accord, Totalement d'accord)
Q20a : Quel serait le seuil de probabilité (en %) donné par le modèle au-dessus duquel je songerais à sevrer l'EER ? (ex: je songerais à sevrer une EER si l'algorithme prédit une sevrabilité avec une probabilité supérieure à x%). (nombre 0-100)
Q20b : Quel serait le seuil de probabilité (en %) donné par le modèle en dessous duquel je ne songerais pas à arrêter l'EER ? (ex: je ne songerais pas à sevrer une EER si l'algorithme prédit une sevrabilité avec une probabilité inférieure à x%). (nombre 0-100)
