# Supplementary material for: Expectations of Intensive Care Physicians Regarding an AI-Based Decision Support System for Weaning From Continuous Renal Replacement Therapy: Predevelopment Survey Study
Source: JMIR Med Inform. 2025 Apr 23;13:e63709. doi: 10.2196/63709 (PMC12043247; doi:10.2196/63709)
Supplement: Multimedia Appendix 4 [file medinform-v13-e63709-s004.pdf]

MULTIMEDIA APPENDIX 4

Expectations of intensive care physicians regarding a decision support tool for weaning from continuous renal replacement therapy: a pre-development survey study

Table S1. Responses to statements on a Likert scale from 1 (strongly disagree) to 5 (totally agree) according to level of training.

Senior physicians correspond to attending physicians, and junior physicians included fellows and junior doctors (final-year residents who have completed their medical thesis and are practicing autonomously under guided supervision according to the French medical education).
Results are presented as median (IQR).
CRRT, continuous renal replacement therapy; ICU, intensive care unit

**Question**	**Junior** (n=33)	**Senior** (n=138)	***P*-values**
**Overview of the problem and current practices**			
Q1: The decision to discontinue CRRT is difficult.	4 (2–4)	3 (2–4)	.06
Q2: In general, when I decide to stop a CRRT, I am certain of my decision.	2 (2–4)	3 (2–4)	.30
**Opinion on clinical decision support systems**			
Q6: I am comfortable with the concept of artificial intelligence.	4 (2, 4)	3 (2, 4)	.07
Q7: I use AI tools in my daily life (outside my clinical practice).	3 (2, 4)	2 (1, 4)	.03
Q8: I use AI tools in my daily clinical practice.	2 (1, 3)	2 (1, 3)	.80
Q9: I think AI tools can help me in my daily clinical practice in intensive care.	4 (4, 4)	4 (3, 4)	.08
Q10: I think AI tools will replace my job in the future.	2 (2, 2)	2 (1, 2)	.3
**Implementation in daily clinical practice**			
Q12: The prediction of weaning from CRRT should be calculated: Continuously	2 (2–3)	3 (2–4)	.2
Punctually, at a specific time (e.g. during morning round)	3 (2–4)	4 (3–4)	.01
Punctually, on demand only	4 (3–4)	3 (2–4)	.05
Q13: Ideally, the decision-support tool should be: A separate software application	3 (2–3)	3 (2–3)	.8
A smartphone/tablet application	3 (2–3)	3 (2–4)	.2
Integrated into the ICU patient management software and visible at the same time as other vital parameters	4 (3–5)	4 (3–5)	.8
Integrated into the ICU patient management software and visible in a separate section (action required to view prediction results)	4 (3–5)	4 (3–4)	.03
Q14: I'm ready to enter variables manually to obtain results from the decision- support tool	4 (4–4)	4 (3–4)	.7
**Real-life operation, willingness to adopt in everyday practice**			
Q16: I think that an AI tool to assist in the decision to wean a patient from CRRT could be an aid in my daily clinical practice.	4 (4–5)	4 (3–4)	.008
Q17: It is important to me that the model gives the percentage of certainty of its prediction before I make the decision to wean a patient off CRRT.	4 (4–5)	4 (3–5)	.08
Q18: It is important for me to understand the criteria on which the model has based its prediction.	5 (4–5)	5 (4–5)	>.9
Q19: I don't think any AI model would influence my decision to wean a patient off CRRT.	2 (2–3)	2 (2–3)	.4

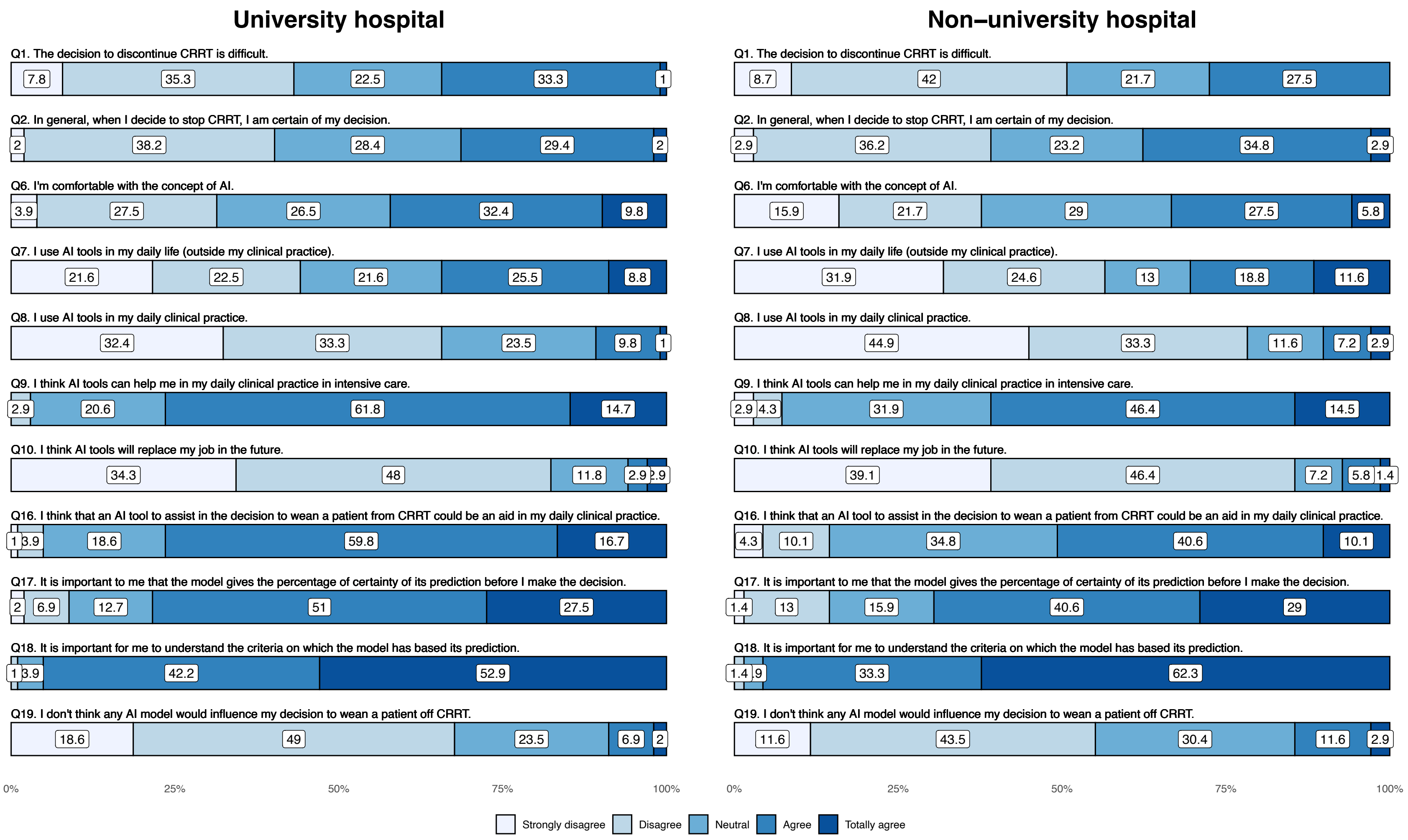

Figure S1. **Clinicians' answers to statements questions according to the academic or non-academic status of the hospital in which they work.**

Results are presented as percentages.
AI, artificial intelligence; CRRT, continuous renal replacement therapy.

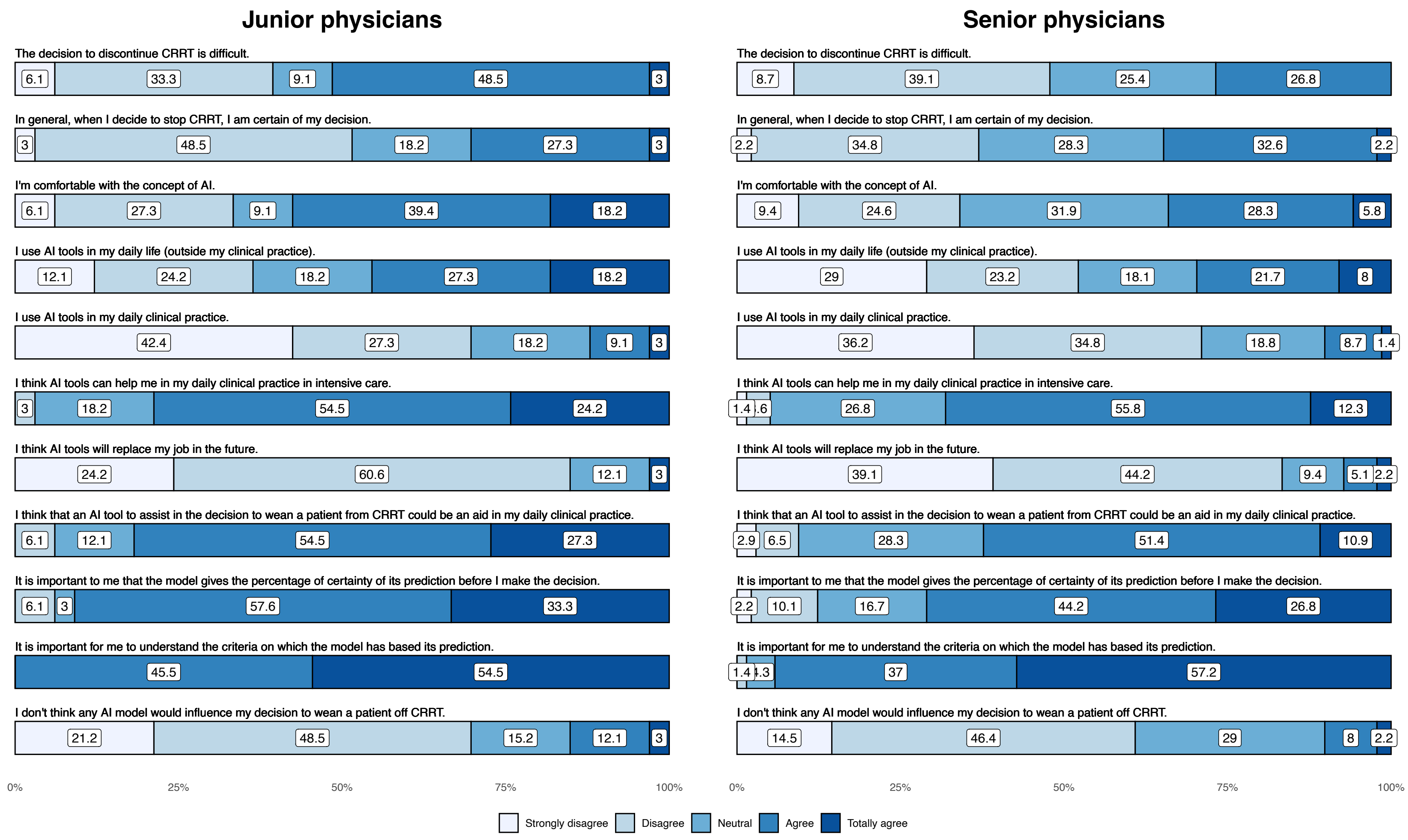

Figure S2. **Clinicians' answers to statements questions according to level of training.**

Results are presented as percentages.
AI, artificial intelligence; CRRT, continuous renal replacement therapy.
